# Development and validation of an automated liquid-liquid extraction GC/MS method for the determination of THC, 11-OH-THC, and free THC-carboxylic acid (THC-COOH) from blood serum

**DOI:** 10.1007/s00216-016-9537-5

**Published:** 2016-04-26

**Authors:** Kirsten Purschke, Sonja Heinl, Oliver Lerch, Freidoon Erdmann, Florian Veit

**Affiliations:** Bonn-Rhein-Sieg University of Applied Science, von-Liebig-Straße 20, 53359 Rheinbach, Germany; Department of Forensic Toxicology, University Hospital Giessen and Marburg GmbH, Institute of Legal Medicine, Frankfurter Straße 58, 35392 Giessen, Germany; GERSTEL GmbH & Co. KG, Eberhard-Gerstel-Platz 1, 45473 Muelheim, Germany

**Keywords:** Cannabinoids, Unconjugated THC-COOH, Liquid-liquid extraction (LLE), Automation, GC/MS

## Abstract

The analysis of Δ^9^-tetrahydrocannabinol (THC) and its metabolites 11-hydroxy-Δ^9^-tetrahydrocannabinol (11-OH-THC), and 11-nor-9-carboxy-Δ^9^-tetrahydrocannabinol (THC-COOH) from blood serum is a routine task in forensic toxicology laboratories. For examination of consumption habits, the concentration of the phase I metabolite THC-COOH is used. Recommendations for interpretation of analysis values in medical-psychological assessments (regranting of driver’s licenses, Germany) include threshold values for the free, unconjugated THC-COOH. Using a fully automated two-step liquid-liquid extraction, THC, 11-OH-THC, and free, unconjugated THC-COOH were extracted from blood serum, silylated with *N*-methyl-*N*-(trimethylsilyl) trifluoroacetamide (MSTFA), and analyzed by GC/MS. The automation was carried out by an x-y-z sample robot equipped with modules for shaking, centrifugation, and solvent evaporation. This method was based on a previously developed manual sample preparation method. Validation guidelines of the Society of Toxicological and Forensic Chemistry (GTFCh) were fulfilled for both methods, at which the focus of this article is the automated one. Limits of detection and quantification for THC were 0.3 and 0.6 μg/L, for 11-OH-THC were 0.1 and 0.8 μg/L, and for THC-COOH were 0.3 and 1.1 μg/L, when extracting only 0.5 mL of blood serum. Therefore, the required limit of quantification for THC of 1 μg/L in driving under the influence of cannabis cases in Germany (and other countries) can be reached and the method can be employed in that context. Real and external control samples were analyzed, and a round robin test was passed successfully. To date, the method is employed in the Institute of Legal Medicine in Giessen, Germany, in daily routine. Automation helps in avoiding errors during sample preparation and reduces the workload of the laboratory personnel. Due to its flexibility, the analysis system can be employed for other liquid-liquid extractions as well. To the best of our knowledge, this is the first publication on a comprehensively automated classical liquid-liquid extraction workflow in the field of forensic toxicological analysis.

**Graphical abstract:**
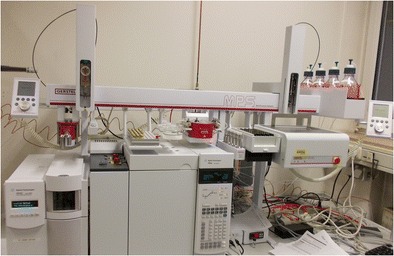
GC/MS with MPS Dual Head at the Institute of Legal Medicine, Giessen, Germany. Modules from *left* to *right*: ^*quick*^Mix (for LLE), wash station, tray 1 (vials for extracts), solvent reservoir, ^*m*^VAP (for extract evaporation), Solvent Filling Station (solvent supply), cooled tray 2 (vials for serum samples), and centrifuge (for phase separation)

GC/MS with MPS Dual Head at the Institute of Legal Medicine, Giessen, Germany. Modules from *left* to *right*: ^*quick*^Mix (for LLE), wash station, tray 1 (vials for extracts), solvent reservoir, ^*m*^VAP (for extract evaporation), Solvent Filling Station (solvent supply), cooled tray 2 (vials for serum samples), and centrifuge (for phase separation)

## Introduction

In the last decades, cannabis was, besides alcohol and tobacco, the most commonly used and also the most controversial discussed (illicit) drug in Germany. Also, driving under the influence of cannabis is becoming a more and more serious issue. Driving license regulations (German law: § 14 FeV Annex 4 no. 9.2) imply that the aptitude for driving a vehicle is not given if regular cannabis consumption can be proven. In such cases, the driving license can be denuded.

In forensic laboratories, evaluation of cannabis consumption is done by urine, blood, hair, or saliva analysis. In hair and urine, cannabinoids can be detected for a longer period of time than in blood. Therefore, in Germany, these matrices are used as part of an abstinence control in medical-psychological assessments [1] to re-obtain the driver’s license. By determining Δ^9^-tetrahydrocannabinol (THC) and its metabolites in blood (serum), laboratories determine the current state of intoxication.

Δ^9^-Tetrahydrocannabinol is the main psychotropic compound in cannabis which is metabolized in the body to 11-hydroxy-Δ^9^-tetrahydrocannabinol (11-OH-THC), 11-nor-9-carboxy-Δ^9^-tetrahydrocannabinol (THC-COOH), and 11-nor-9-carboxy-Δ^9^-tetrahydrocannabinol glucuronide (THC-COOgluc). In the first phase of metabolism in which oxidation, reduction, and/or hydrolysis take place, THC is converted to the still pharmacologically active hydroxy metabolite 11-OH-THC. Afterwards, 11-OH-THC is oxidized in the liver to the psychotropically inactive metabolite THC-COOH. This is followed by the enzymatically catalyzed conjugation with glucuronic acid to form the phase II metabolite THC-COOgluc [2]. After 30–60 min, the concentration of the glucuronide exceeds that of the free carboxylic acid [3]. Glucuronidation makes the molecule more hydrophilic; thus, the excretion via urine is improved and THC-COOgluc constitutes the main metabolite in urine.

Up to a limit of 1 μg/L THC in serum, it is considered that the driving ability is not impaired (German law: § 24 a (2) StVG). Moreover, there are limits for the slowly degrading (half-life 6 days), inactive phase I metabolite THC-COOH [4]. This analyte serves to evaluate the consumption behavior of a driver. It must be ensured that only the free THC-COOH (free carboxylic acid) concentration is determined and this is not erroneously increased by coextraction and/or cleavage of the conjugated phase II metabolite THC-COOgluc [5].

Originally, gas chromatographic separation coupled to mass spectrometric detection with electron impact ionization using the single ion monitoring mode (GC/EI-SIM-MS) was employed to quantify cannabinoids in serum, plasma, or whole blood [6–10]. Moreover, GC/MS with positive chemical ionization has been employed [11]. In order to gain selectivity, GC/MS/MS methods have been developed, using electron impact [12] or negative chemical ionization [13]. Two-dimensional GC/MS has been used as an alternative to improve selectivity [14, 15]. Analyte derivatization is predominantly carried out with silylation reagents [9, 11, 12, 14, 15] and sometimes with fluorinated compounds [6, 13] or methylation reagents [7, 8, 10]. In recent years, more and more liquid chromatography tandem mass spectrometry (LC/MS/MS) methods were described in the literature [16–23]. For sample preparation, both solid-phase extraction (SPE) [7–12, 14–16, 19–21] and liquid-liquid extraction (LLE) techniques [6, 13, 17, 18, 22] have been applied. In routine forensic analysis, oftentimes, liquid-liquid extraction with *n*-hexane/ethyl acetate (9/1, *v*/*v*) is employed [6, 18, 22]. Under certain conditions, THC-COOgluc may be coextracted and partly cleaved during the derivatization step forming the THC-COOH derivative and thus leading to erroneously elevated THC-COOH analysis values [20, 24].

Manual sample preparation normally comprises numerous steps representing a significant workload for laboratory staff with exposure to potentially toxic solvents and reagents, and it means that errors are more likely to occur. Therefore, complete automation of the analysis is preferable. Fully automated and partly automated sample preparation, mainly for SPE, is applied in some forensic laboratories. Benchtop systems using standard SPE cartridges mimic the manual SPE workflow [9, 10]. Complete automation of sample preparation and analysis is possible with online SPE systems where the SPE cartridge is integrated into an LC flow path and the cartridge is automatically exchangeable [19, 25] or not [16, 20]. Complete automation of SPE, evaporation, derivatization, and injection into a chromatographic system are possible as well [26]. Automation of liquid-liquid extraction is more difficult. It can be realized in the form of, e.g., single-drop microextraction [27] or in 96-well format micro-tubes [28].

The aim of this study was the development of a comprehensively automated analysis method for the determination of THC, THC-OH, and free THC-COOH in blood serum basing on a previously developed manual method.

## Materials and methods

### Solvents, reagents, and standards

All analytes and deuterated analogs were certified standards. (−)-Δ^9^-THC (1 g/L in methanol), (−)-Δ^9^-THC-d_3_, (±)-11-hydroxy-Δ^9^-THC, (±)-11-hydroxy-Δ^9^-THC-d_3_, (±)-11-nor-9-carboxy-Δ^9^-THC, (±)-11-nor-9-carboxy-Δ^9^-THC-d_3_, (+)-11-nor-9-carboxy-Δ^9^-THC-glucuronide, and (±)-11-nor-9-carboxy-Δ^9^-THC-d_3_-glucuronide (each 0.1 g/L in methanol) were purchased from Cerilliant (Round Rock, USA). Blood serum samples were taken from real forensic cases of the Institute of Legal Medicine (Giessen, Germany). Internal quality control (QC) samples consisted of drug-negative serum given by voluntary donors from the institute spiked with THC, 11-OH-THC, and THC-COOH. Two QCs were prepared, the first one containing 1 μg/L THC and 11-THC-OH and 10 μg/L THC-COOH and the second one containing 30 μg/L THC and 11-THC-OH, and 300 μg/L THC-COOH. External quality control samples were certified reference materials (ACQ Science GmbH, Rottenburg-Hailfingen, Germany). An internal standard solution was prepared at 5 μg/L THC-d_3_ and 11-OH-THC-d_3_ as well as 50 μg/L THC-COOH-d_3_ in methanol. Calibration solutions were prepared either by diluting the certified standards in methanol or by spiking drug-negative serum.

All solvents were of analytical grade and purchased from VWR (Darmstadt, Germany). For liquid-liquid extraction, a mixture of *n*-hexane and ethyl acetate (9/1, *v*/*v*) was prepared. *N*-Methyl-*N*-(trimethylsilyl) trifluoroacetamide (MSTFA) for silylation was from Macherey-Nagel (Dueren, Germany). Derivatization was carried out either with pure MSTFA in the manual workflow or with a mixture of MSTFA/ethyl acetate (3/2, *v*/*v*) in the automated workflow.

### Instrumentation—manual liquid-liquid extraction workflow

For manual extraction, a Vortex-Genie 2 (Scientific Industries, New York, USA) was employed. The extraction tubes were put into an EBA 200 centrifuge (Hettich, Tuttlingen, Germany) for phase separation. After withdrawal of the organic phase, it was evaporated in a heating block (Barkey, Leopoldshöhe, Germany) with ten nitrogen-streamed vial positions (Linde, Pullach, Germany). Derivatization was carried out in a laboratory oven (Memmert, Schwabach, Germany).

The extracts were analyzed with an 6890 GC/5973N MSD (Agilent Technologies, Waldbronn, Germany). A 7683 autosampler was applied for injection into a hot split/splitless inlet (Agilent Technologies). Analytes were separated on an Optima 5 HT (30 m × 0.25 mm, 0.25 μm film thickness, Macherey-Nagel) column.

### Instrumentation—automated liquid-liquid extraction workflow

**Fig. 1:**
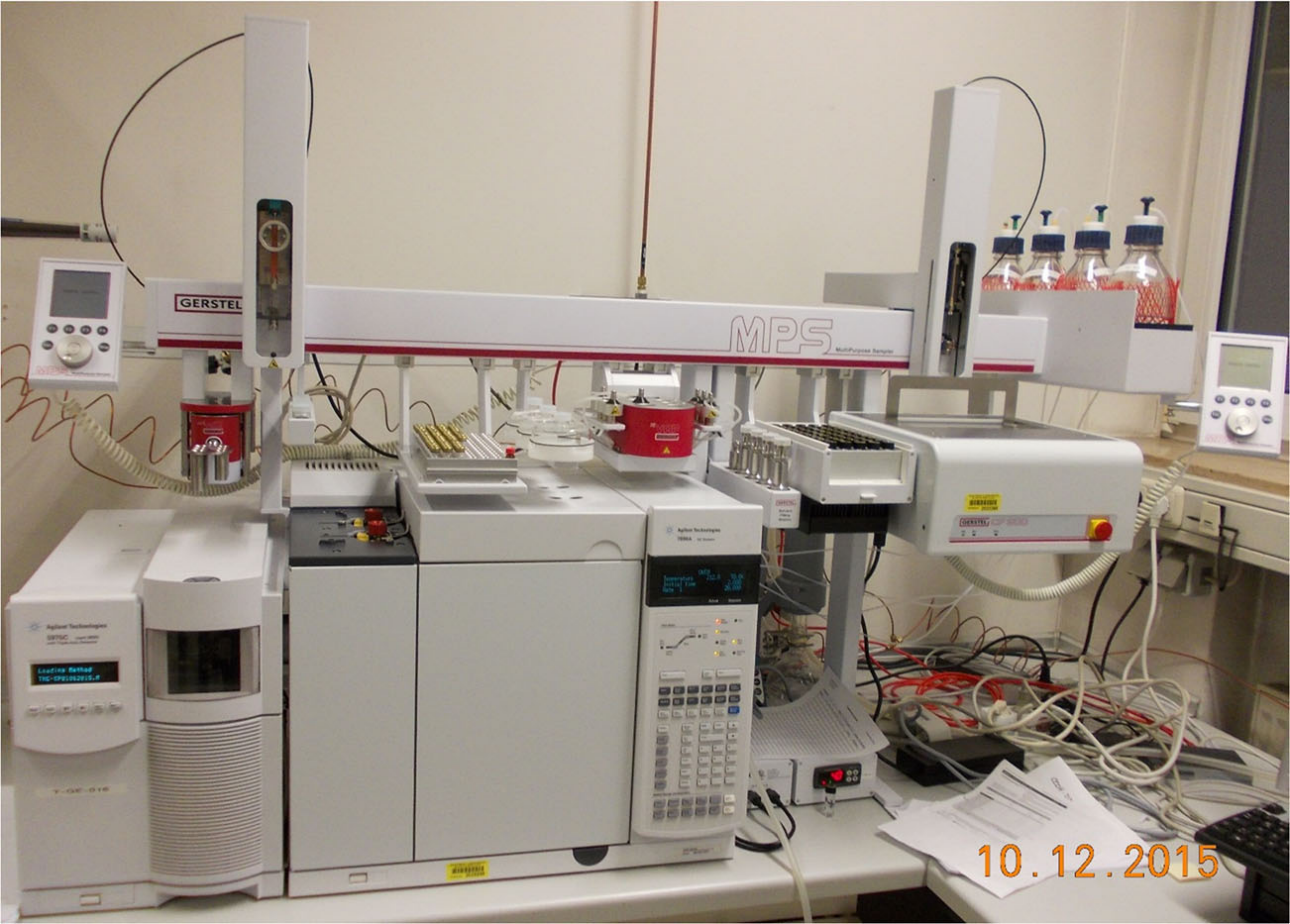
GC/MS with MPS Dual Head at the Institute of Legal Medicine, Giessen, Germany. Modules from *left* to *right*: ^*quick*^Mix (for LLE), wash station, tray 1 (vials for extracts), solvent reservoir, ^*m*^VAP (for extract evaporation), Solvent Filling Station (solvent supply), cooled tray 2 (vials for serum samples), and centrifuge (for phase separation)

Comprehensive automation of sample preparation was based on a MultiPurpose Sampler MPS (GERSTEL, Muelheim, Germany; see Fig. 1) which is a flexible platform with numerous available modules. It carried two syringes, a 1 mL for sample preparation steps and a 10 μL for sample injection into a 7890A GC/5975C MSD (Agilent Technologies) equipped with a DB-5-MS (30 m × 0.25 mm, 0.25 μm film thickness, Agilent Technologies) column. The MPS was equipped with a centrifuge (CF 200), a module for evaporation of solvents under controlled vacuum and temperature (Multi Position Evaporation Station, ^*m*^VAP), a ^*quick*^Mix for sample extraction, and a Solvent Filling Station (SFS 2, all GERSTEL) for solvent supply. All sample preparation steps were performed in septum-sealed vials with magnetic screw caps enabling transport by the MPS. The system was controlled via Maestro software (version 1.4.33.3, GERSTEL) which was integrated into the ChemStation chromatography data system (version E.02.02 SP1, Agilent Technologies). With this software configuration, all sample preparation steps could be combined freely and were included into the GC/MS analysis method.

### Analysis methods—manual liquid-liquid extraction workflow

One milliliter of blood serum was mixed with 50 μL of the deuterated internal standard solution. Liquid-liquid extraction was carried out with 5 mL *n*-hexane/ethyl acetate (9/1, *v*/*v*) for 1 min on a vortex shaker. After centrifugation (RCF = 2500×*g*), the organic phase was withdrawn; the remaining aqueous sample was acidified with 100 μL of 1 M HCl (resulting pH 4–5), extracted, and centrifuged again to obtain a second extract. The combined organic phases were evaporated to dryness under nitrogen at 48 °C. For derivatization, the dry sample was silylated with 50 μL MSTFA in an oven at 80 °C for 30 min. Finally, 2 μL of the sample was injected into the split/splitless inlet of the GC/MS at 280 °C. Separation was achieved with a constant helium flow of 1.3 mL/min and the following temperature program: 70 °C for 2 min, rate 20 °C/min to 250 °C for 4 min, rate 20 °C/min to 300 °C for 17 min. Mass spectrometric detection was done in single ion monitoring (SIM) mode (see Table 1).

**Table 1:** Target and qualifier ions for analytes and deuterated internal standards

	Target (*m*/*z*)	Qualifier 1 (*m*/*z*)	Qualifier 2 (*m*/*z*)
THC	386	371	303
THC-d_3_	389	374	306
11-OH-THC	371	474	459
11-OH-THC-d_3_	374	477	462
THC-COOH	371	473	488
THC-COOH-d_3_	374	476	491

### Analysis methods—automated liquid-liquid extraction workflow

For automation, 0.5 mL of blood serum was manually mixed with 25 μL of the deuterated internal standard solution in a 4-mL vial. All further steps were carried out fully automated by the MPS. The sample was extracted with 1.5 mL *n*-hexane/ethyl acetate (9/1, *v*/*v*) for 3 min in the ^*quick*^Mix and centrifuged for phase separation at RCF = 2000×*g* for 7 min. The supernatant was taken into another vial and evaporated to dryness under controlled vacuum (1 min at 70 kPa, 3.5 min at 4 kPa) and temperature (65 °C) and shaking (3.1 Hz) in the evaporation station while the residue was acidified with 50 μL of 1 M HCl (resulting pH 4–5). After acidic extraction, again with 1.5 mL *n*-hexane/ethyl acetate (9/1, *v*/*v*), the organic phase was separated, transferred to the same extract vial, and evaporated again until dryness was reached. A mixture of 50 μL MSTFA/ethyl acetate (3/2, *v*/*v*) was added to the dry residue, the vial was shortly shaken, and 2 μL was injected into the hot split/splitless inlet (derivatization at 280 °C) of the GC/MS. For practical reasons, the temperature program was changed compared to the manual method, since there was a second GC column for a different application installed in the same GC oven which could not withstand temperatures higher than 260 °C. Separation was performed with a constant helium flow of 1.6 mL/min and the following temperature program: 70 °C for 2 min, rate 20 °C/min to 240 °C for 5 min, rate 20 °C/min to 250 °C for 6 min, rate 20 °C/min to 260 °C for 5 min. Mass spectrometric detection was done in single ion monitoring (SIM) mode (Table 1).

For calibration, both methanolic solutions and spiked serum samples were employed. Methanolic calibration solutions and the methanolic control samples were handled analogously to the extracts. Calibration with solvent standards is often used in forensic toxicology and is accepted by the Society of Toxicological and Forensic Chemistry (GTFCh) if equivalence with matrix calibration can be proven. The manual and automated analysis methods were validated according to GTFCh guidelines with the help of the software Valistat (version 1.0) from Arvecon (Walldorf, Germany).

Analysis sequences comprised 20 real cases and four control samples (negative, low concentration, high concentration, and external). According to GTFCh recommendations, a blank injection of pure derivatization reagent was done before every real sample.

## Results and discussion

### Method development

A dual-stage liquid-liquid extraction method for the determination of THC, 11-OH-THC, and free THC-COOH in blood serum was developed. Considering the different p*K*a values of the analytes, the first extraction was carried out at the native pH of the blood serum (around pH 7). At this pH, mainly THC and 11-OH-THC which are non-charged were extracted into the organic phase. In the second extraction step, after acidification of the residual sample to pH 4–5, free THC-COOH was extracted and separated from THC-COOgluc. At this pH, THC-COOgluc (p*K*a ∼3.2 [29]) predominantly (about 90 %) is present deprotonated (anionic) and thus cannot pass into the organic phase. Free THC-COOH (p*K*a ∼4.2 [29]), however, is protonated and non-charged (about 50 %) and can be extracted into the organic phase. Maintaining the pH between 4 and 5 is crucial for the second extraction step. Major deviations can lead to glucuronide cleavage [3] or coextraction of THC-COOgluc which forms silylated THC-COOH during the derivatization step. Eventually, both effects result in erroneously elevated THC-COOH analysis values [20, 24].

For confirming that the developed analysis method does not result in elevated THC-COOH values, the following experiment was conducted: 12 samples of blank blood serum were spiked with equal amounts of THC-COOH and the internal standard THC-COOH-d_3_. Six of these samples were additionally spiked with THC-COOgluc. The peak area ratio THC-COOH/THC-COOH-d_3_ was calculated for all analyses. On average, the samples additionally spiked with THC-COOgluc showed a 1.5 % higher ratio proving that only negligible amounts of THC-COOH were formed from THC-COOgluc by the sample preparation steps (see Table 2). In that respect, recommendations for interpretation of analysis values in medical-psychological assessments requiring the analysis of free THC-COOH only without interferences by THC-COOgluc were fulfilled.

**Table 2:** Comparison of THC-COOH/THC-COOH-d_3_ ratios for blank serum spiked at 50 μg/L with THC-COOH and THC-COOH-d_3_ only and blank serum additionally spiked at 50 μg/L with THC-COOgluc. Analysis results for THC-COOH are not biased by THC-COOgluc addition

Blank serum spiked with THC-COOH and THC-COOH-d_3_			Blank serum additionally spiked with THC-COOgluc			Difference of ratios
Response THC-COOH	Response THC-COOH-d_3_	Ratio	Response THC-COOH	Response THC-COOH-d_3_	Ratio	
7348	6907	1.064	7395	6564	1.127	+0.063
7896	6884	1.147	8856	7833	1.131	−0.016
6854	6320	1.085	7606	6683	1.138	+0.054
7967	7176	1.110	6503	5468	1.189	+0.079
7409	6179	1.199	7935	7192	1.103	−0.096
	Average	1.121			1.138	+0.017

The developed two-step liquid-liquid extraction was successfully and completely automated using a MultiPurpose Sampler equipped with different modules (e.g., shaker, centrifuge, evaporator). Some analysis parameters were improved or needed to be adapted for automation.

The sample volume could be reduced from 1 to 0.5 mL serum. Also, the volume of the extraction solvent for each extraction step was reduced from 5 to 1.5 mL *n*-hexane/ethyl acetate (9/1, *v*/*v*). This enabled the use of 4-mL vials for extraction, extending the sample capacity to 50 samples per sequence.

Centrifugation time and needle penetration depth for the extract withdrawal step needed to be optimized carefully since it is crucial to prevent contamination of the final extract by residual serum. Also, optimal evaporation parameters had to be found out ensuring on the one hand that the extract is completely evaporated before derivatization and on the other hand that the analytes are not exposed to heat in the ^*m*^VAP longer than necessary.

**Fig. 2:**
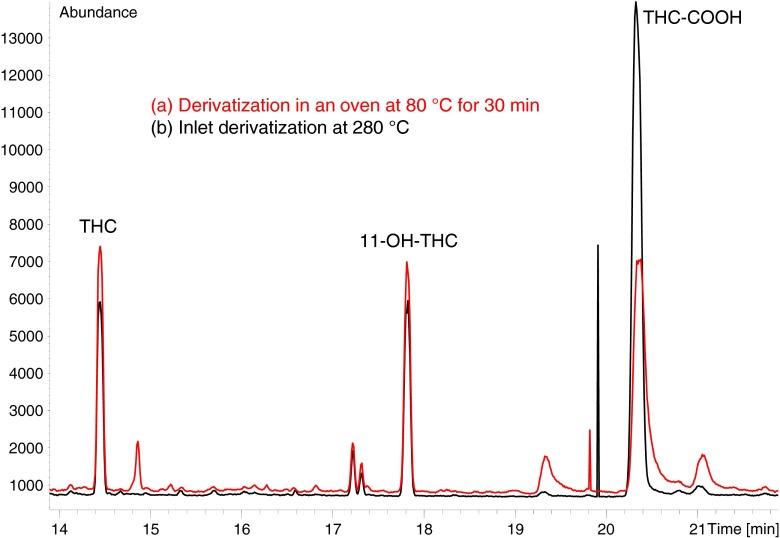
Chromatograms of serum samples spiked with 4 μg/L THC and THC-OH and 40 μg/L THC-COOH after extraction and derivatization with MSTFA in an oven for 30 min at 80 °C (**a**) and derivatization with MSTFA/ethyl acetate (3/2, *v*/*v*) in the GC inlet at 280 °C (**b**)

Analyte derivatization can be done during injection into the hot split/splitless inlet with a mixture of MSTFA/ethyl acetate (3/2, *v*/*v*) instead of pure MSTFA. In comparison to the 30-min derivatization step in an oven, this saves time and increases throughput while analytical performance is maintained. Chromatograms of the inlet derivatization showed less and smaller background peaks than chromatograms received with 30 min derivatization in an oven (see Fig. 2). The reduction of MSTFA volume percentage enhances the lifetime of the analytical column and the autosampler syringe according to former experiences with silylation reagents in our laboratory.

### Method validation

Requirements of the validation according to GTFCh guidelines [30] have been met at each point for both the manual and automated methods. Only validation data for the automated method are discussed here.

Selectivity and specificity were checked by analyzing six different serum samples spiked with opiates and benzodiazepines as they could be present in real samples as well. No interferences from the spiked drugs or the matrix were recorded at the retention time of analytes and internal standards of interest.

Linearity of calibration was proven up to 35 μg/L for THC and 11-OH-THC and up to 350 μg/L for THC-COOH respectively. A calibration with standards in methanol was equivalent to a matrix calibration and therefore was employed for analyzing real samples. Precision, accuracy, and extraction efficiency data can be found in Table 3. All precision and accuracy values were below the acceptable value of 15 %. The average extraction efficiency was 110 % ranging from 103 to 121 %. Limits of quantification (LOQ) and detection (LOD) for the respective compounds are listed in Table 4. Most importantly, the limit of quantification for THC was far below 1 μg/L, being an important limit in driving under the influence of cannabis cases in Germany and other countries. No carry-over was detected for any compound when analyzing a blank serum sample after a serum sample spiked at the highest calibration level (35 μg/L for THC and 11-OH-THC and 350 μg/L for THC-COOH). Freeze/thaw stability was confirmed by three freezing and thawing cycles with spiked QC samples of low (1.5 μg/L for THC and 11-OH-THC and 15 μg/L for THC-COOH) and high (12.5 μg/L for THC and 11-OH-THC and 125 μg/L for THC-COOH) concentrations.

**Table 3:** Excerpt of validation data of the automated analysis method according to GTFCh guidelines

Analyte	Concentration [μg/L]	Repeatability: RSD_r_ [%]	Time-different intermediate precision: RSD_(T)_ [%]	Accuracy: bias [%]	Extraction efficiency [%]
THC	1	7.8	6.1	2.1	109
	30	2.1	8.5	0.7	103
11-OH-THC	1	8.3	8.1	4.1	120
	30	1.8	8.7	−0.3	103
THC-COOH	10	2.4	6.3	3.0	121
	300	0.9	12	0.9	104

**Table 4:** Limits of detection (LOD) and limits of quantification (LOQ) for the automated method

	THC [μg/L]	11-OH-THC [μg/L]	THC-COOH [μg/L]
LOD	0.3	0.1	0.3
LOQ	0.6	0.8	1.1

**Fig. 3:**
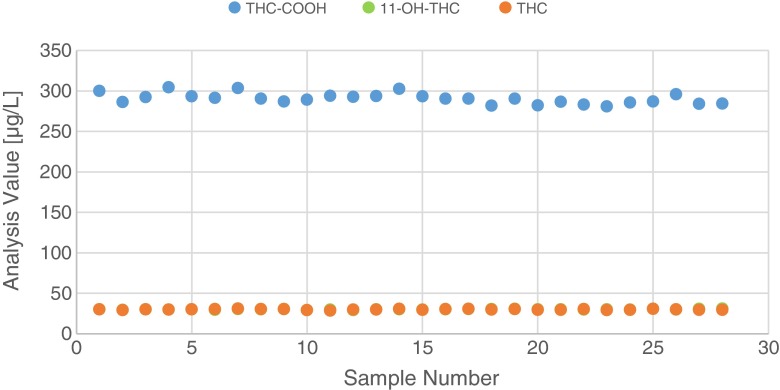
Analysis values for a sequence of 28 blank serum samples spiked with 30 μg/L THC and 11-OH-THC and 300 μg/L THC-COOH and 300 μg/L THC-COOgluc stored on a cooled tray at 4 °C. No increase of the THC-COOH value by cleavage of THC-COOgluc can be observed during the sequence run time of more than one day. Note that data points for 11-OH-THC analysis values are overlapped by data points for THC analysis values since these analytes were spiked at the same concentration

Furthermore, stability of the samples on the autosampler tray during an analysis sequence was evaluated. For this, a serum pool was spiked with 30 μg/L THC and 11-OH-THC and 300 μg/L THC-COOH and THC-COOgluc. Aliquots of 0.5 mL were put into autosampler vials. The first series of samples was processed immediately; the second series was stored overnight on the autosampler tray at room temperature. Average analysis values for THC and 11-OH-THC were concordant. For THC-COOH, an increase of around 10 % after storage could be observed which can be explained by THC-COOgluc degradation and the formation of THC-COOH. When storing the serum samples at 4 °C on a cooled tray and running a sequence of 28 samples (runtime more than 1 day), neither an increase nor a decrease of analysis values could be observed for all analytes (see Fig. 3). Cooling of samples is essential for running analysis sequences over a long period of time.

LOQs of the developed automated method lie well within published LOQs for GC/MS(/MS) [6, 8, 9, 11, 13] and LC/MS/MS [16–18, 20, 21] methods. In the literature, these were between 0.5 and 1 μg/L for THC and 11-OH-THC and between 0.8 and 4.3 μg/L for THC-COOH. A sophisticated 2D-GC/MS method yielded LOQs of 0.125 μg/L for THC and THC-COOH and 0.25 μg/L for 11-OH-THC respectively [14]. Extraction efficiencies and recoveries mentioned in the literature were mainly lower than in the present study which might be caused by differences in the way of sample spiking. As in our application—except for THC-COOH at 300 μg/L—relative standard deviations (precision) in the literature were below 10 %. A round robin test has been passed successfully with the automated method and analysis values for external control samples laid inside the acceptable concentration ranges (Table 5).

**Table 5:** Analysis results of a successfully passed round robin test (two samples) and external control samples employing the comprehensively automated method

Analyte	Analysis value [μg/L]	Target value [μg/L]	Acceptable range [μg/L]
THC	6.7^a^	6.0	3.9–8.1
	3.9^a^	4.4	2.8–5.9
	1.1^b^	1.1	0.6–1.6
	8.5^b^	9.5	6.4–12
	14^b^	20	14–26
11-OH-THC	3.1^a^	2.6	1.6–3.7
	2.5^a^	2.6	1.5–3.6
	1.1^b^	1.1	0.6–1.5
	4.7^b^	5.0	3.2–6.8
	9.8^b^	9.8	6.6–13
THC-COOH	75^a^	71	54–88
	64^a^	63	47–78
	8.4^b^	10	6.9–14
	73^b^	71	54–88
	141^b^	136	107–165

^a^Round robin test sample

^b^External control sample

### Comparison of manual and automated methods

In order to check the newly validated methods (manual and automated), 20 real samples from the Institute of Legal Medicine in Giessen and various external controls were analyzed and compared.

**Fig. 4:**
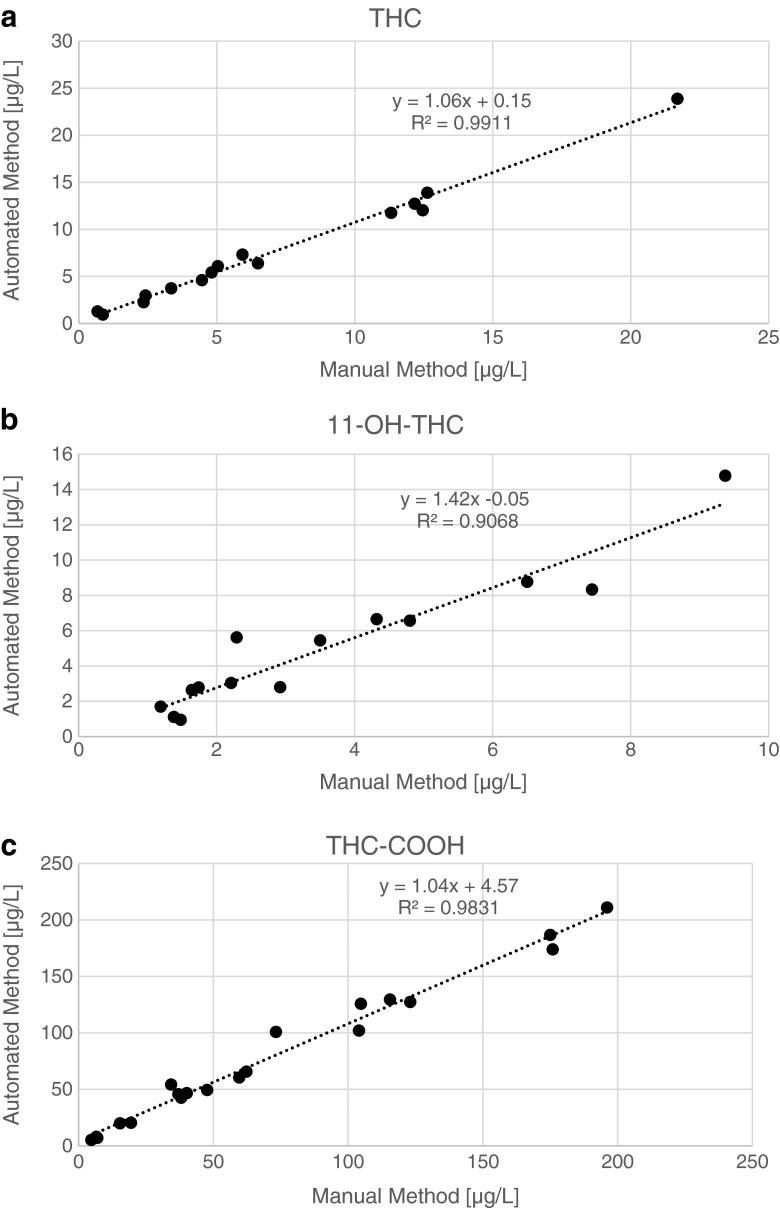
Comparison of analysis results for manual and automated sample preparation of 20 real samples: **a** THC, **b** 11-OH-THC, **c** THC-COOH. Equations resulting from linear regression and coefficients of determination are shown. Not all samples included the analytes in concentrations above the respective limit of quantification

Considering the fact that comparative analyses of real samples were conducted on different instruments, the concentration values fit quite well especially for the most important analytes THC and THC-COOH (see Fig. 4a, c). For 11-OH-THC, the deviation of analysis values was slightly larger but acceptable (see Fig. 4b). All analysis values for 11-OH-THC fall in the lower concentration range where 11-OH-THC reveals the highest bias and lowest repeatability of all compounds for both the automated (Table 3) and manual methods (not shown). This may explain the larger deviations for 11-OH-THC in comparison to THC and THC-COOH.

After successful validation, both the automated and manual methods for the determination of THC and metabolites in serum are suitable for use in forensic toxicological analysis. It is noteworthy that postmortem samples cannot be analyzed ruggedly with the automated method because of the possible formation of a gel during the extraction step which cannot be separated by the standard centrifugation method.

## Conclusions

One manual and one fully automated analysis method for THC, 11-OH-THC, and free, unconjugated THC-COOH in blood serum were developed and successfully validated according to GTFCh guidelines. Analysis values were concordant, and both methods ensure that only the free THC-COOH concentration is determined and the analysis value is not erroneously increased by THC-COOgluc coextraction and/or cleavage. Regarding LOQs, extraction efficiencies, and precision, both developed methods correspond well to methods from the literature. The LOQ for THC of 1 μg/L required for driving under the influence of cannabis cases in Germany can be reached, and the method can be employed in that context.

To the best of our knowledge, this is the first publication on a comprehensively automated classical liquid-liquid extraction workflow in the field of forensic toxicological analysis. Also, the employed analysis system including shaker, centrifuge, and evaporator modules is mentioned for the first time in the literature.

These are the main achievements and benefits:

- The automated method saves manual work and reduces the risk of human errors. This makes analysis quality projectable and more independent of the human factor.
- By overlapping sample preparation and GC/MS run, the comprehensively automated method has a throughput of 22 samples per day compared to 20 samples per day with the manual workflow. Eighty-three minutes is needed for a single analysis (including sample preparation, GC/MS analysis, and the required blank analysis), meaning that results are available 83 min after putting a single sample onto the autosampler.
- The automated method can be employed for all types of sera except postmortem samples.
- The automated system proved to be rugged and is employed in our daily routine in the institute.
- The analysis system can be used for other matrices (e.g., saliva or urine) and other liquid-liquid extraction workflows also outside the field of forensic toxicology and is therefore of general interest.
- Other liquid-liquid extraction workflows in our laboratory are actually under consideration for automation.
